# Editorial: Understanding convergent evasion mechanisms in cancer and chronic infection: implications for immunotherapy

**DOI:** 10.3389/fimmu.2024.1450555

**Published:** 2024-07-01

**Authors:** Hakim Echchannaoui, Matthieu Perreau, Hansjörg Schild, Matthias Theobald

**Affiliations:** ^1^ Department of Hematology and Medical Oncology, University Cancer Center (UCT), University Medical Center (UMC) of the Johannes Gutenberg University, Mainz, Germany; ^2^ German Cancer Consortium (DKTK), Partner site Frankfurt/Mainz, Mainz, Germany; ^3^ Division of Immunology and Allergy, Lausanne University Hospital, University of Lausanne, Lausanne, Switzerland; ^4^ Institute of Immunology, UMC of the Johannes Gutenberg University, Mainz, Germany; ^5^ Helmholtz Institute Translational Oncology (HI-TRON), Mainz, Germany

**Keywords:** immunotherapy, tumor microenvironment, chronic infection, immune evasion, metabolism, cutting edge technologies

The complex interactions between the innate and adaptive immune systems function to recognize and clear pathogens or transformed cells, but inefficient interactions can result in harmful immunologic responses including chronic infections and the development of cancer.

In this research topic, we compile recent developments in the understanding of the common and novel immune/therapy related evasion mechanisms in cancers and in diverse chronic viral infections, and discuss the complex interplay between chronic infection/inflammation and cancer. We also outline cutting-edge technologies to characterize immune responses at a tissue/single cell level, highlighting therapeutic strategies to manipulate the tumor microenvironment (TME) for effective immunotherapy, and discussing advances in artificial intelligence (AI) models for personalized cancer immunotherapy.

## 1 Immune/therapy-related evasion in chronic infection

Immune checkpoint inhibitors (ICI) have emerged as breakthrough generation of therapeutics in immunotherapy. However, treatment with ICI can be associated with immune-related adverse events (irAEs). In this research topic, Zeng et al. explored the impact of PD-1 inhibitor in combination therapy on the incidence of HBV reactivation (HBVr) and irAEs. The study showed that PD-1 inhibitor combinational therapy may have therapeutic potential for chronic HBV (CHB) infection, however HBVr observed in a group of patients has been attributed to immunosuppression induced by activation of suppressive cells by PD-1 inhibitors, calling for more attention using ICI in patients with CHB.

The drivers behind the generation of an effective CD8 response in conditions where antigen presentation is altered, such as during viral infections, remain elusive. Holtappels et al. investigate cytomegalovirus (CMV)-specific CD8 T cells priming in the clinically relevant setting of post-hematopoietic cell transplantation (HCT) immune reconstitution, taking advantage of recombinant murine-CMV viruses producing the central immune evasion protein m152, which inhibits MHC class I presentation. Their findings suggested that direct antigen presentation by infected APCs may be the primary pathway responsible for CD8 T cells priming in CMV infection during hematopoietic reconstitution after HCT.

## 2 Bridging chronic viral infection/inflammation and cancer

Altered metabolic activity in the TME is a hallmark of cancer. Extracellular Adenosine (Ado) in the TME leads to profound immunosuppression by downregulating the activation and effector functions of different immune cells and, promoting M2-type macrophage polarization and tolerogenic dendritic cell differentiation to favor tumor growth. Chen et al. have reviewed the different and convergent mechanisms of how Ado-induced immune suppression, initially induced in inflammation, can in the course of chronic and prolonged inflammation lead to tumor formation and outgrowth.

Studies reporting on the role of cytotoxic CD4^+^ T cells (CD4^+^ CTL) are increasing in the context of chronic (and acute) viral infections and, recently also in cancer. In this context, Malyshkina et al. provided a thorough and comprehensive review on the complex and dynamic role of CD4^+^ CTL in diverse chronic viral infections and solid tumors, shedding light on their potential in immunotherapy and vaccine development.

## 3 Novel immune-related evasion pathways in non-solid cancer (AML)

In rare cases, Acute Myeloid Leukemia (AML) can regress in the absence of therapies, however the underlying mechanisms remains poorly understood. Here, Koedijk et al. present a unique case with immune dysregulation and long-lasting regression of a (pre)leukemic clone in the absence of therapy. Thorough molecular and immunological analyses revealed immune-mediated bone marrow features associated with this regression, suggesting immune-mediated control of the (pre)leukemic clone before it developed into overt AML. Moreover, the authors identified additional genetic alterations at AML diagnosis that may have contributed to immune escape of the (pre)leukemic clone.

## 4 TME-mediated immune evasion in solid cancer

ICI remain effective in the treatment of hepatocellular carcinoma (HCC), however drug resistance and relapse are often associated with poor prognosis. In this research topic, Chen et al. have extensively reviewed the mechanisms underlying TME-mediated immunosuppression in HCC, describing the complex interaction of the immune microenvironment in particular with dysfunctional metabolism and gut microbiota, and discussing therapeutic strategies to manipulate the TME in favor of more effective immunotherapy. Casari et al. have reviewed in depth the current knowledge on the critical role of hepatic macrophages and platelets in liver fibrosis and HCC progression, shedding light on their complex interplay and their contribution to the formation of an immune suppressive tumor milieu in HCC and other solid cancers. Therefore, modulating macrophages and platelets crosstalk may represent a new therapeutic approach for HCC.

## 5 Cutting-edge technologies to characterize immune responses

Because the TME plays a pivotal role in cancer initiation and progression, in-depth analyses of such immune landscape and validated experimental protocols to isolate and characterize immune cells from the TME are essential. In this context, this research topic features a series of methodological manuscripts aiming to provide a “toolbox” for immunologists interested in the study of immune responses at a tissue level.

The paper from Eich et al., illustrates a step-by-step protocol to induce a colorectal cancer model in mice and to analyze the inflammatory infiltrate associated with tumor development. The authors took advantage of a clinically relevant model of cancer development, using azoxymethane and dextran sodium sulphate to induce tumorigenesis, and describe how to retrieve tumor mass, measure tumor burden, and isolate a single-cell suspension of leucocytes to be analyzed by flow cytometry.

In addition to flow cytometry, other sequencing-based single-cell technologies can investigate immune cells landscape with unprecedented resolution. Two additional methodological papers included in this research topic aim to provide guidance to design and interpretate single-cell sequencing experiments. In the first one, Braband et al. illustrate how to isolate high-quality nuclei from CD4 T cells infiltrating different murine tissues, how to create libraries for single cell Assay for Transposase-Accessible Chromatin-sequencing (ATAC-Seq) using the 10x Genomics platform, and how to analyze sequencing results on a bioinformatic pipeline. In the second one, Nedwed et al. provide a protocol to isolate CD4 T cells from murine tissues and multimodally investigate transcriptome and TCR sequencing by scRNA/TCR-seq. The authors show how to isolate cells and how to perform sample barcoding by adding cell surface antibodies coupled with distinct oligonucleotide barcodes. This approach allows the subsequent multiplexing of different samples from simultaneous sequencing, greatly reducing the costs and allowing the high-throughput, high-dimensional exploration of anti-tumor immune responses.

## 6 Strategies to improve cancer immunotherapy

Immunotherapy has dramatically improved the outcome of patients with solid tumors and lymphatic neoplasms. In AML, these approaches have been far less successful. The relatively low mutational burden and the absence of cancer-specific antigens in AML has hindered the creation of effective immune-based strategies for cure. Hence, a novel use of already available treatment options may prove useful in selected clinical conditions. In this context, this research topic features a review from Rausch et al. exploring the possible applications of combined epigenetic targeting and immunotherapy to enhance antigen presentation on tumor cells and reduce proliferation of cancer clones in AML. The clinical implications of this treatment strategy, as well as the ongoing clinical trials exploring this option are herein reviewed.

Bartneck et al. evaluated a non-invasive immunization platform DIVA as a therapeutic vaccination method. Using the murine MC38 tumor model, the authors showed that DIVA resulted in transient tumor control followed by an immune evasion phase. Deep characterization of the TME using high dimensional flow cytometry and scRNA-seq of tumor-infiltrating leukocytes, identified a CCR2^+^ PD-L1^+^ monocyte population with immunosuppressive properties, as novel potential target to enhance efficacy of tumor vaccination and counteract tumor immune escape.

## 7 Advances in machine learning models for cancer immunotherapy

Targeting cancer neoantigens for precision immunotherapy is a rapidly advancing field. Combining high-throughput sequencing data with deep learning algorithms and AI has strengthen the traditional methods for neoantigen prediction, by allowing a rapid processing of large-scale data and a more accurate identification of therapeutically relevant neoantigens. In this review article, Bulashevska et al. provide a detailed overview of the current state-of-the-art techniques in neoantigen prediction, exploring the strengths and limitations of a broad range of AI-driven approaches. The work highlights the current challenges of AI for its clinical implementation in cancer immunotherapy.

## Author contributions

HE: Conceptualization, Writing – original draft, Writing – review & editing. MP: Writing – original draft, Writing – review & editing. HS: Writing – original draft, Writing – review & editing. MT: Conceptualization, Writing – original draft, Writing – review & editing.

